# Capturing Electrocardiogram Signals from Chairs by Multiple Capacitively Coupled Unipolar Electrodes

**DOI:** 10.3390/s18092835

**Published:** 2018-08-28

**Authors:** Zhongjie Hou, Jinxi Xiang, Yonggui Dong, Xiaohui Xue, Hao Xiong, Bin Yang

**Affiliations:** State Key Laboratory of Precision Measurement Technology and Instruments, Department of Precision Instrument, Tsinghua University, Beijing 100084, China; hou-zj15@mails.tsinghua.edu.cn (Z.H.); xiangjx16@mails.tsinghua.edu.cn (J.X.); xxh17@mails.tsinghua.edu.cn (X.X.); xiong-h13@mails.tsinghua.edu.cn (H.X.); yangbin_1006@163.com (B.Y.)

**Keywords:** unipolar electrode, smart chair, phase space reconstruction, capacitively coupled ECG

## Abstract

A prototype of an electrocardiogram (ECG) signal acquisition system with multiple unipolar capacitively coupled electrodes is designed and experimentally tested. Capacitively coupled electrodes made of a standard printed circuit board (PCB) are used as the sensing electrodes. Different from the conventional measurement schematics, where one single lead ECG signal is acquired from a pair of sensing electrodes, the sensing electrodes in our approaches operate in a unipolar mode, i.e., the biopotential signals picked up by each sensing electrodes are amplified and sampled separately. Four unipolar electrodes are mounted on the backrest of a regular chair and therefore four channel of signals containing ECG information are sampled and processed. It is found that the qualities of ECG signal contained in the four channel are different from each other. In order to pick up the ECG signal, an index for quality evaluation, as well as for aggregation of multiple signals, is proposed based on phase space reconstruction. Experimental tests are carried out while subjects sitting on the chair and clothed. The results indicate that the ECG signals can be reliably obtained in such a unipolar way.

## 1. Introduction

Sensor technologies are widely used in our everyday lives. Wearable devices such as electronic clothes [1], watches [2], necklaces [3], etc. that can conveniently obtain basic physiological parameters of the human body have become an important research area [4,5]. However, people are reluctant to carry and wear these devices all the time. In order to address these limitations of wearable devices, sensors could be embedded on physical objects or appliances used frequently in daily life [6], and thus help improve the intelligence of our living environment. Different sensing solutions for the development of smart environments have been reported. Lee et al. [6] developed a smart bed for long-term heart rate monitoring. Conductive textiles are embedded on the bed and used as capacitively coupled electrodes. Braun et al. [7] introduced a CapFloor system for indoor localization and fall detection. The system is based on a grid array of sensing electrodes placed below a floor covering. A smart toilet system was developed by La et al. [8]. The toilet is equipped with an Arduino board and various sensors to measure health conditions such as weight, body temperature, pulse, blood oxygen and blood pressure.

Office workers, the elderly and students spend most of the day sitting on chairs [9,10]. According to the chair usage research [10], 55% of office workers spend more than 9 h a day siting on chairs. Therefore, with sensors embedded on a regular chair, there are valuable opportunities to monitor and analyze daily activities and health parameters of people. For example, Hesse et al. [11] developed a chair with integrated force sensitive resistors, radar sensor and actuators. It is able to improve the subject’s well-being by offering qualified fitness training, relaxation mode and assistive functions. Kumar et al. [12] constructed a chair with force sensitive resistors to estimate breathing rate and emotion-based activities.

Heart rate variability (HRV) is highly related to stress, anxiety, diabetes, hypertension, fatigue, and depression symptoms [13,14,15]. Devices that can measure electrocardiogram (ECG) signals, especially the HRV, are becoming powerful tools for self-monitoring in daily life. Baek et al. [16] developed a chair that can monitor multiple biological signals including ECG, ballistocardiograph (BCG), and photoplethysmogram (PPG). By simultaneously measuring the signals, blood pressure can be estimated by the pulse arrival time (PAT) that calculated from the ECG and PPG signals. Similar work can be found in [17,18]. Singh et al. [19] installed six capacitive ECG electrodes (cECGs) into an automotive seat. One channel of ECG signal is obtained by differentially amplifying the outputs of a pair of the cECGs. The ECG signal with the best quality is selected by a frequency domain-based switching logic. Choi et al. [20] attached four cECGs (EPIC Sensor) on the backrest of a chair. One pair of the cECGs is used to measure the ECG signal, and another pair is used to obtain the human body motion information. It is reported that the motion artifacts can be reduced by such an approach. To our knowledge, most of the reported systems pick up the ECG signal in a differential measurement way, i.e., one ECG signal is obtained from the outputs of a pair of electrodes. However, for the chair-based ECG signal acquisition system, the signal quality is related to the individual body shape, sitting posture, the clothing thickness and so on. Because of the imbalance of the two electrode-skin coupling impedances, signal quality will fluctuate, and sometimes the ECG signal may even be ruined by the motion artifacts during such differential measurement procedure. A typical example can be found in [21] (Section 4). The unipolar measurement, on the other hand, picks up the biopotential signal from one single measurement electrode [22]. Koichi Mizukami et al. [23] recorded two-channel 24-h Holter ECG with unipolar leads (V1 and V2) and aimed to estimate the ST segment during the daily life. Gargiulo et al. [22,24,25,26] introduced a nine-channel unipolar ECG system. The front-end circuits are designed based on the voltage supply bootstrap technology. Albert et al. [27] recorded 12-lead unipolar ECG by using only one limb of the Wilson Central Terminal. The measurement results indicate that unipolar ECG signals are highly correlated (r > 9) with standard leads. Up to our knowledge, most of the reported works use the conventional wet Ag/AgCl or dry electrodes in their systems. Their research aims to reduce the signal redundancy with unipolar measurement.

A unipolar approach for ECG signal acquisition is experimentally tested in this paper. Different from the conventional differential measurement, the capacitively coupled sensing electrodes in our approaches operate in the unipolar mode. The outputs of each sensing electrodes are amplified and sampled separately. Since the unipolar electrodes are independent, the measured ECG signals will not affect each other, problems caused by impedance imbalance can be overcome. Four unipolar electrodes are mounted on the backrest of a regular chair to get four-channel ECG signals. The experimental results indicate that such an approach gives robust performance to sitting postures and body types of the tested subjects.

## 2. Materials and Methods

### 2.1. Experimental Setup

**Figure 1:**
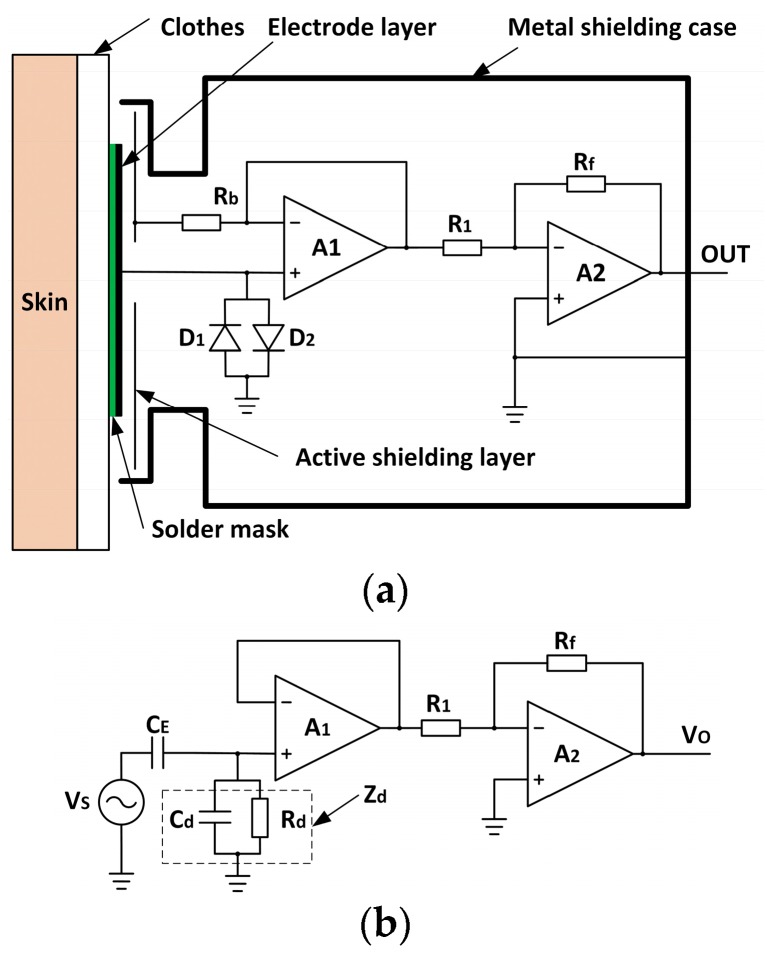
Sensing electrode: (**a**) Electrode structure; (**b**) Circuit model.

Figure 1a shows the schematic diagram of the sensing electrode. The physical substrate of the electrode is a standard multilayer printed circuit board (PCB). The electrode layer is the first conductive layer of the PCB, which is covered by the solder mask for electrical insulation. The electrode layer and the human skin form an approximately parallel plate capacitor to sense the potential variation on the skin. The rear side of the electrode layer is surrounded by the active shielding layer. The entire electrode is covered by a metal shielding case (aluminum). The active shielding layer and the metal shielding case are used to prevent the electrode from unwanted interference noises. The electrode circuit consists of two operation amplifier (Op-Amp), LMP7721 (A1) and OPA2333 (A2). The A1 is configured as a unity gain voltage buffer. Two anti-parallel diodes ($D_{1,2}$, IN4148) are used as ultra-high value resistors (beyond 1 TΩ) to minimize the input noise, and to prevent the static electricity interference. When the static electricity of the human body is accumulated greater than the built-in potential (0.6 V for IN4148), one of the diodes will turn on and release it. The A2 is used to amplify the signal to the appropriate range. 

**Figure 2:**
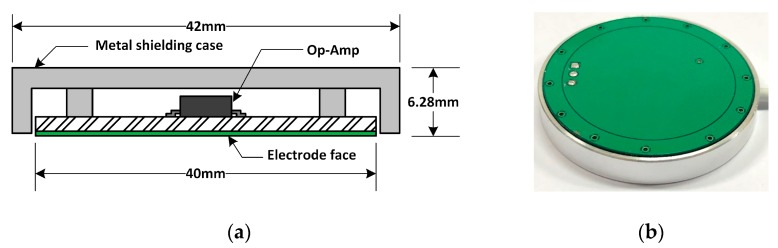
Picture of the sensing electrode: (**a**) Structure diagram; (**b**) The appearance of the electrode.

Figure 1b depicts a simplified, generic circuit model for the sensing electrode. The tested ECG signal can be thought as an AC voltage source $V_{s}$. $Z_{d}$ ($C_{d}//R_{d}$) is the equivalent impedance of $D_{1,2}$. $C_{E}$ is the coupling capacitance between the electrode layer and skin:

(1) $$C_{E}=\frac{\varepsilon_{0}\varepsilon S}{d}$$

where $\varepsilon_{0}$ is the vacuum dielectric constant ($\varepsilon_{0}=8.85\times 10^{-12}\;F/m$), ε is the dielectric constant of the coupling medium, S is the effective area of the electrode layer, and d is the effective distance between the electrode and the human skin. Since the medium between the electrode and the human skin is the clothes, the dielectric constant of the clothes is approximately the dielectric constant of the air (ε ≈ 1) [28]. The structure of the sensing electrode can be seen in Figure 2. The effective area is 12.56 ${\mathrm{cm}}^{2}$. In our experimental cases, the effective distance *d* is about 0.3 mm according to cotton shirt thickness. Therefore, the value of *C_E_* is about 37 pF by Equation (1). The transfer function of the front circuit around A1 in Figure 1b is *H*_1_:

(2) $$H_{1}=\frac{Z_{d}}{Z_{E}+Z_{d}}\;\mathrm{where}\;Z_{d}=\frac{R_{d}}{1+j\omega R_{d}C_{d}}$$

The transfer function of the inverting amplifier A2 is

(3) $$H_{2}=-\frac{R_{f}}{R_{1}}$$

Because the entire circuit is cascade-connected by these two parts, and the transfer function of the electrode is

(4) $$H=\frac{V_{O}\;}{V_{S}}=H_{1}\cdot H_{2}=\frac{j\omega R_{d}C_{E}}{1+j\omega R_{d}\left(C_{E}+C_{d}\right)}\cdot \left(-\frac{R_{f}}{R_{1}}\right)$$

The equivalent capacitance of the diode is about 1 pF, which is far less than the coupling capacitance ($C_{d}\ll C_{E}$). Therefore, the transfer function can be simplified as:

(5) $$H\approx \frac{j\omega R_{d}C_{E}}{1+j\omega R_{d}C_{E}}\cdot \left(-\frac{R_{f}}{R_{1}}\right)$$

**Figure 3:**
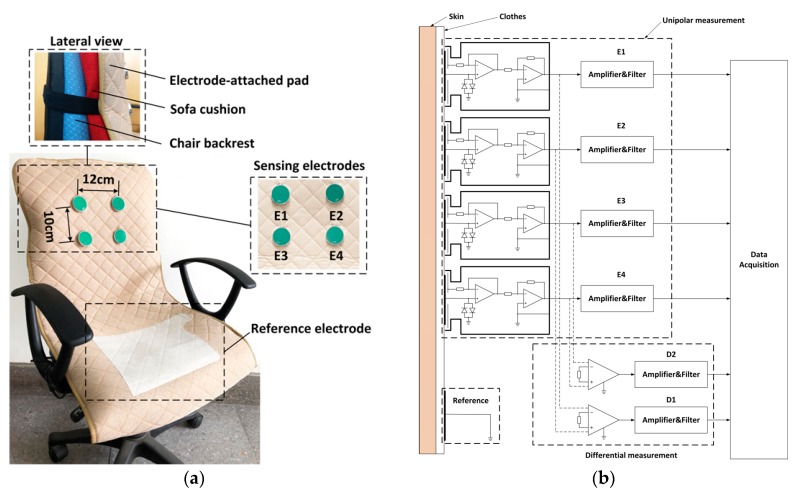

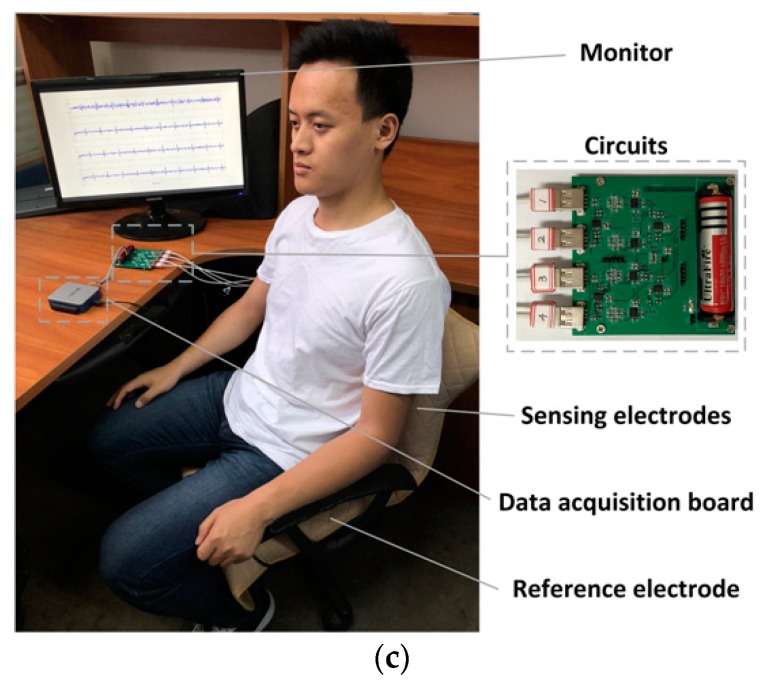
The ECG signal acquisition system: (**a**) Picture of the electrode positions; (**b**) Signal acquisition system; (**c**) Prototype of the system.

The prototype of the experimental system is shown in Figure 3. Four sensing electrodes are attached on a thin pad (0.5 cm) called the electrode-attached pad. A sofa cushion with thickness 10 cm is inserted between the electrode-attached pad and the backrest of a regular chair (Figure 3a). 

**Figure 4:**
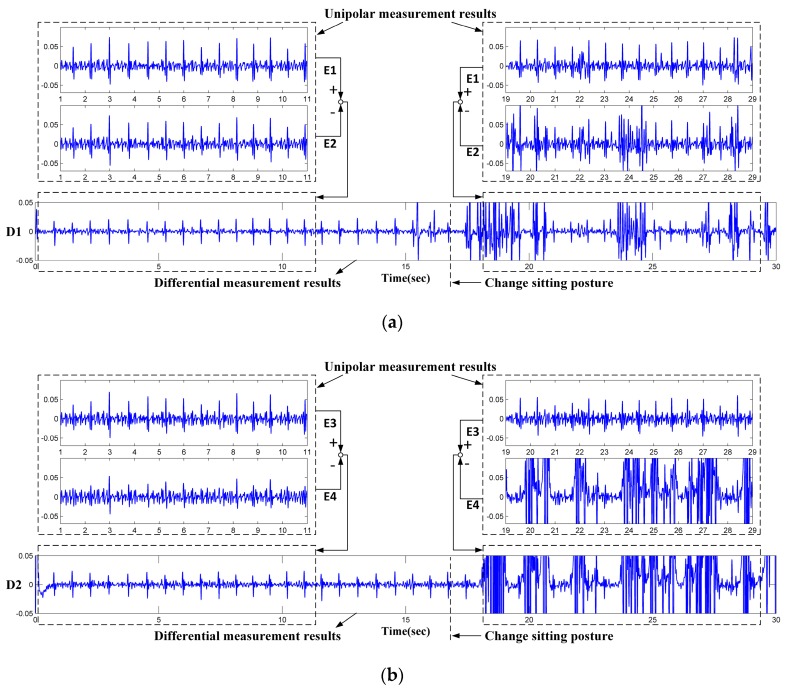
ECG signals obtained by differential measurement and unipolar measurement (Y-axis: measured amplitude in volts): (**a**) ECG signal results of E1, E2 and D1; (**b**) ECG signal results of E3, E4 and D2.

The vertical and the horizontal distance between the sensing electrodes is 10 cm and 12 cm, respectively. The sensing material of the reference electrode is made of conductive fabric and mounted on the seat of the chair. The signal acquisition system is shown in Figure 3b. In order to compare the performance of the two measurement approaches, both the unipolar (solid line) and differential (dashed line) measurement circuits are implemented. Two channel of the differential measurement are acquired by the circuits given in the lower portion of Figure 3b. Four channel of the unipolar signals are directly obtained from the four unipolar electrodes (the upper portion of Figure 3b). The analog amplifier and filter circuits in Figure 3b are the same, i.e., all the analog signals are sampled after a high-pass filter (cutoff frequent is 0.3 Hz), a notch filter (center frequency is 50 Hz) and a low-pass filter (cutoff frequent is 200 Hz). The filtered ECG signals are sampled and converted to digital signal at 1000 Hz sampling frequency by the data acquisition board (DAQ, USB-6003, National Instruments, Austin, TX, USA). In order to verify the validity of the unipolar approach, all the six-channel signals in Figure 3b are sampled simultaneously with one subject (male, 1.75 m and 76 kg) sitting on the chair as seen in Figure 3c. The subject was wearing a cotton T-shirt (about 0.3 mm thickness). At the beginning of the test, the subject is asked to sit on the chair and adjust the sitting posture until satisfied ECG signals can be observed from D1 and/or D2. The posture adjustment is not necessary in practical measurement cases. It is designed here simply to ensure that clear QRS complexes can be observed from at least one of the differential ECG signals (D1/D2) at the beginning of the recordings. Then the signals are continuously recorded while the subject is asked to sit there as still as possible. After about 16 s, the subject is asked to change his sitting posture slightly. The recorded signals are given in Figure 4. It can be seen that the ECG signals can be observed clearly from the differential measurement (D1 and D2) before 16 seconds. After that, D1 and D2 become very noisy because of sitting motions. On the other hand, as shown in the upper part of Figure 4a,b clear ECG signals can also be observed from one channel of unipolar signals (E1 and E3). We attribute this phenomenon to the imbalance of the original unipolar signals. As can be seen in Figure 4b, even within the duration of 0–16 s while satisfied ECG signals can be observed from D2, the E4 gives relatively noisy signals compared with that of E3. After 16 s, the E4 is ruined by sitting motions and poor differential measurement results are obtained by D2, even though clear ECG signals can still be observed from E3. Similar results can be observed from Figure 4a. Just as reported works in refs [16,17,18,19,20], when the system is working in the differential measurement mode, the signal quality of the obtained ECG signals may be unstable. Three main reasons could explain this phenomenon as follows:

(1) The electrodes are fixed on the chair. During the measurement, the subjects may perform slight changes in sitting postures and do some daily work. The capacitive electrode is hard and can slip over the clothes, the output signal is therefore susceptible to artifacts because of the poor contact and charging effects [29]. In addition, since the biopotential is picked up in a capacitive-coupling way, the electrode placed on different positions will result in different ECG signal quality [30].
(2) Most clothes are soft and flexible. It is easy to produce random cloth folds when subjects are sitting on the chair with their back leaning on the backrest. The uneven distribution of clothes wrinkles may cause inconsistent distances between differential measurement electrodes and the skin.
(3) Because the shape of the human back is irregular, during the measurement, the electrodes at different positions are subjected to different pressures. The pressure on the electrodes affects the contact conditions and therefore the impedances between the electrodes and the skin. The quality of the measured ECG signal is then be affected [31].

Based on the abovementioned experiments, it can be concluded that the capacitively coupled ECG signal acquisition system with unipolar measurement should be more suitable for the chair-based ECG applications. Compared with the conventional differential measurement, under the same number of sensing electrodes, the unipolar measurement can obtain more signal channels and can provide more information. Furthermore, because the sensing electrodes in the unipolar measurement are mutually independent, the ECG signals measured by the unipolar electrodes do not affect each other. These advantages enable the proposed system to accommodate to uneven thickness of clothes and different kinds of sitting postures.

### 2.2. Signal Acquisition and Processing

**Figure 5:**
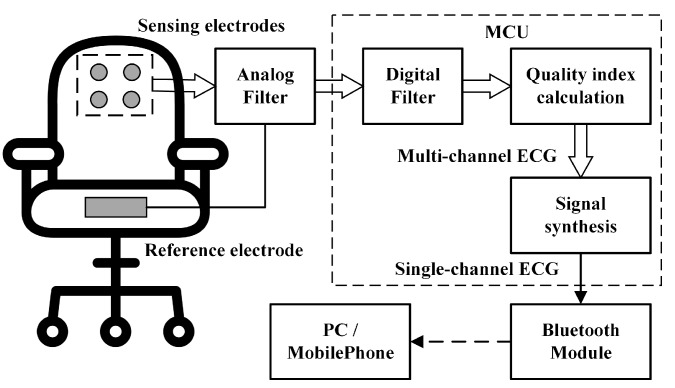
Systemic diagram.

The working principle of the proposed ECG signal acquisition system is shown in Figure 5. The original ECG signals are measured by the sensing electrodes in a unipolar way. After the analog filters (see Section 2.1), the signals are sampled and processed locally in the microcontroller unit (MCU) system. The digital filter is implemented by three filters in series, including a 50 Hz notch filter, a four-order low-pass filter (cutoff frequency is 40 Hz) and a two-order high-pass filter (cutoff frequency is 0.3 Hz). The digital filter is used for eliminating power-line interference, low-frequency baseline wandering and high-frequency electromyographic noise. The quality indexes of the multi-channel are calculated. Based on the quality indexes, multi-channel ECG signals are synthesized into single-channel ECG signal. After that, the synthetic signals are transmitted to the PC or a mobile phone wirelessly via Bluetooth for future processing and displaying.

As mentioned above, due to the different sitting posture and body size, not all the channels could get reliable ECG signals. An effective algorithm for evaluating the signal quality is needed. Frequency domain features of the ECG signals are generally used to evaluate the signal quality [19,32,33]. 

**Figure 6:**
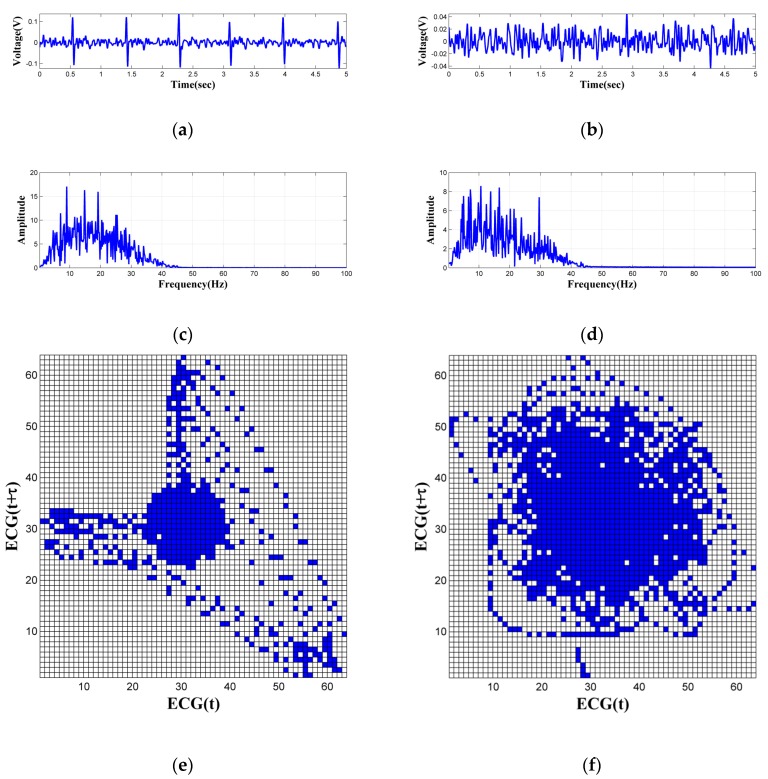
The frequency domain features of ECG signals and phase portraits boxes visualized: (**a**) The clear ECG signals; (**b**) The noisy ECG signals; (**c**) The frequency domain features of the clear ECG signals; (**d**) The frequency domain features of the noisy ECG signals; (**e**) The gridded phase portraits of the clear ECG signals; (**f**) The gridded phase portraits of the noisy ECG signals.

Figure 6a,b show two segments of ECG signals measured from one subject. The corresponding frequency domain distribution of these signals are given in Figure 6c,d, respectively. It is obvious that the ECG features in Figure 6a are clear than that in Figure 6b. More precisely, the measurement results of Figure 6b is totally disturbed by noise. However, both of the frequency domain distribution of these signals are concentrated in 5–30 Hz. It is difficult to distinguish between the clear and noisy ECG signals based on frequency domain features. This is because that the frequency of the QRS complex ranges between 4 and 20 Hz [34], and most of the low-frequency noise are located within this range. This phenomenon is common in capacitively coupled ECG signals. Therefore, the ECG signal evaluation method that based on frequency domain features is not suitable for our system. An index for evaluating the ECG signal quality is proposed based on phase space reconstruction. For a one-dimensional time series $X,X=\left(x_{1},x_{2},x_{3},\cdots ,x_{N}\right),$ m-dimensional delay vectors $p\left(i\right),p\left(i\right)=\left(x_{i},x_{i+\tau },x_{i+2\tau },\cdots ,x_{i+\left(m-1\right)\tau }\right)$ can be obtained by the embedding method [35]. m is the mapping dimension of the phase space, and τ is the time delay. m=2 and τ=20 ms are satisfactory to extract the feature of the ECG signal [36]. Based on our previous work, the two-dimensional phase space is partitioned into $2^{M}\times 2^{M},\;M=6$ grids and each grid is called a box. This gridding procedure can be done by numerically truncating the high M-bits from the raw digital data. Figure 6e,f show that the gridded phase portraits of the ECG signals in Figure 6a,b. The gridded phase portraits of the clear ECG signals (Figure 6e) have a relatively regular distribution and only small parts of the phase space are filled. However, the gridded phase portraits of the noisy ECG signals (Figure 6f) is irregular and almost distributed over the entire phase space. Since the phase portraits of the noisy ECG signals will fill more area of the phase space, a quality index, which is defined as Rate by Equation (6), is used as a parameter for signal evaluation:

(6) $$Rate=\frac{N_{p}}{N_{t}}$$

$N_{p}$ is the number of the boxes that visited by the phase portraits of the ECG signals. $N_{t}$ is the total number of the boxes in the phase space. A sliding window is applied for processing the recorded ECG signals. The window length is set to be two seconds in our cases. This setup is based on the phenomenon that the heart rate of healthy people is generally not slower than 40 beats per minute. A 2 s-length sliding window is sufficient to contain a complete heartbeat cycle. The sliding step is 1.5 s with 0.5 s overlapped. The Rate of the ECG signals in the sliding window is calculated.

**Figure 7:**
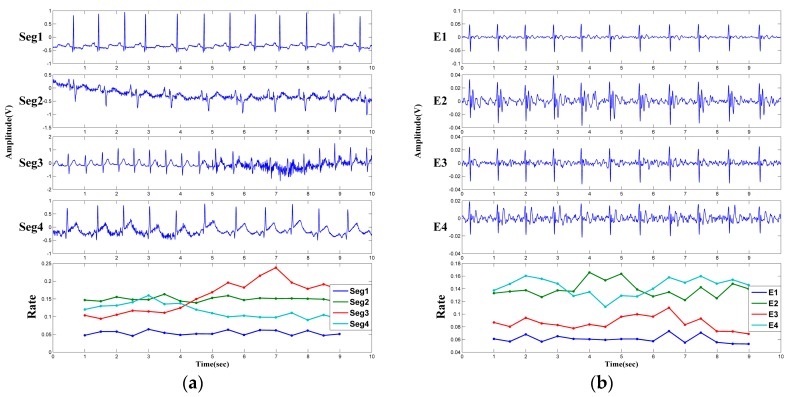
ECG signals and the corresponding Rate values: (**a**) ECG signals obtained from MIT-BIH Database; (**b**) ECG signals measured by the proposed system.

In order to evaluate the availability of the above mentioned signal quality index, simulation is firstly carried out with four segments of ECG signal selected from the MIT-BIH Database [37]. The selected segments (indicated as seg1–seg4 respectively) are shown in Figure 7a. Segment-1 (No. 100) is clear and almost no noise interferences can be observed. In Segment-2 (No. 108), the QRS complexes are not obvious, and there are noise interferences between R-R peaks. Segment-3 (No. 215) and Segment-4 (No. 228) give similar performances, that is, the SNR of one-half of the segment is higher than that of another half of the segment. The calculated Rate values are shown in the bottom of Figure 7a. Because the SNR of the Segment-2 is lower, its Rate keeps a higher value. As for the Segment-3, the Rate values of the front part of the ECG signal are lower, and the Rate values increase with the decreasing signal quality. Similar results can be observed for Segment-4. One experimental example is given in Figure 7b. The four-channel ECG signals measured from one subject are indicated as E1–E4 respectively. As can be seen, the signal quality of E1 and E3 is better than that of E2 and E4. Correspondingly, the Rate values of E1 and E3 are relatively small. These two experimental results show that the quality index can reflect the quality change of the ECG signals.

The calculated Rate values are used for signal synthesis shown in Figure 5. Let $X_{k},X_{k}={\left[x_{k1},x_{k2},\cdots ,x_{kN}\right]}^{T}$ be the signals measured from channel-k (1≤k≤K), and K is the total number of the channels (K=4 in our cases). For each $X_{k}$, the $Rate_{k}$ has been calculated, and the weight value w for each channel is defined as:

(7) $$w_{k}=e^{-{\left(\frac{Rate_{k}}{\sigma }\right)}^{2}}/\sum_{k=1}^{K}e^{-{\left(\frac{Rate_{k}}{\sigma }\right)}^{2}}$$

The scaling factor σ modulates the distribution of weights. If σ≫Rate, then for all the channels, the weight is almost equal to 1/K. A very small σ could make a great difference between the weights. In our cases, the σ is set to 0.3. The weighted data are given by

(8) $$WX=\left[w_{1}\cdot X_{1},w_{2}\cdot X_{2},\cdots ,w_{K}\cdot X_{K}\right]$$

The synthetic result $X_{syn}$ is used as the processed ECG signal as follows.

(9) $$X_{syn}=\sum_{k=1}^{K}w_{k}\cdot X_{k}$$

**Figure 8:**
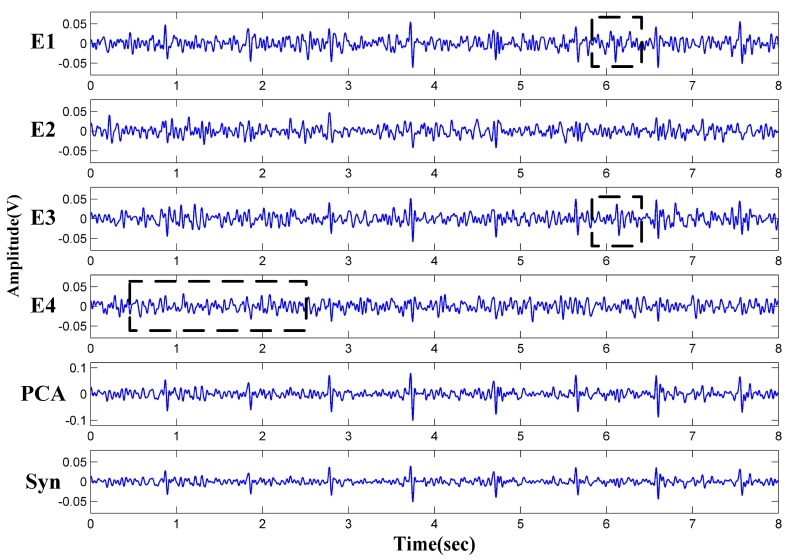
Measured ECG signals and synthetic results.

One example of the signal synthesis is given in Figure 8. All the measured unipolar signals are disturbed by noises (E1–E4). In E4, the QRS features is hard to observe between 0.5 and 2.5 s. As for the E1 and E3, the abnormal peak between 5.8 and 6.3 s may be treated as the normal heart beat. The synthetic results $X_{syn}$ are indicated as Syn in Figure 8. It can be seen that the missing waveforms and false R-peaks in E1, E3 and E4 disappeared in Syn. Moreover, the noise between the R-R intervals are also suppressed, providing a more reliable and clear ECG waveform. As a comparison, the results calculated by Principal Component Analysis (PCA) [38], that the original four channels data are reduced to one dimension based on the maximum eigenvalue, are also given in Figure 8. Obviously, the synthetic method can obtain a similar result compared with that by PCA. Since the singular value decomposition (SVD) computation in PCA algorithm is avoided, less computation cost is needed by this synthetic method. In order to evaluate the processing time of the algorithms, the 8 s ECG signals in Figure 8 are processed on MATLAB (CPU: Intel-i7 6700, Memory size: 16 GB). After a few repetitions, the average execution time for the PCA algorithm is 1.58 ms. On the other hand, the synthetic method only takes 0.14 ms.

## 3. Evaluation and Results

### 3.1. Backrest Softness and Electrode Positions

**Figure 9:**
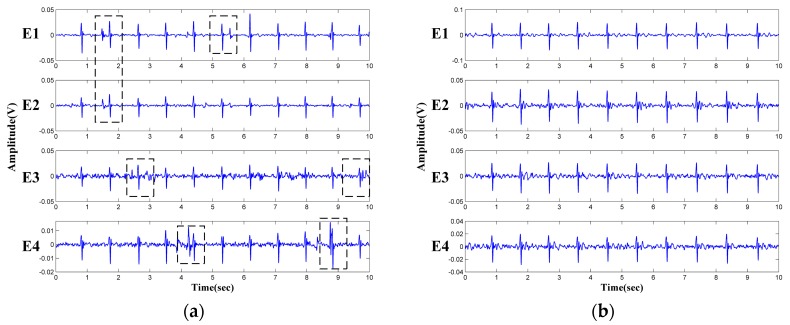
ECG signals obtained under different backrest: (**a**) Firm configuration; (**b**) Soft configuration.

The contact conditions between the sensing electrodes and the human body are the most important factor for capacitively coupled ECG measurements. Because the sensing electrodes are mounted on the backrest of the chair, two backrests with different softness are experimentally tested. In the first case, which is indicated as firm configuration, the electrode-attached pad is placed directly on the chair. The tested backrest is relatively firm. In the second case, which is indicated as soft configuration, a sofa cushion with thickness 10 cm is inserted between the electrode—attached pad and the backrest of the chair as shown in Figure 3a. This configuration makes the tested backrest relatively softer. A subject is asked to sit on the chair and keep motionless. The thickness of the subject’s clothes is 0.3 mm. The measurement results are shown in Figure 9. In the first configuration, although the QRS waveforms can be observed, the signals are more susceptible to motion artifacts (see the black rectangular frames). On the other hand, as can be seen from Figure 9b, the obtained ECG signals are clear, and the periodic QRS waveforms are clearly visible. Under the same thickness of the clothes, the signal quality of the firm backrest configuration is not as good as that of the soft backrest configuration. This is because the soft backrest allows the sensing electrodes to better fit the body shape. When there are slightly motions of the human body, the soft backrest can act as a buffer. Such phenomena may explain why the reported ECG-chairs are implemented on soft backrests [16,18,21]. Therefore, the soft backrest configuration is adopted in the followed experiments.

**Figure 10:**
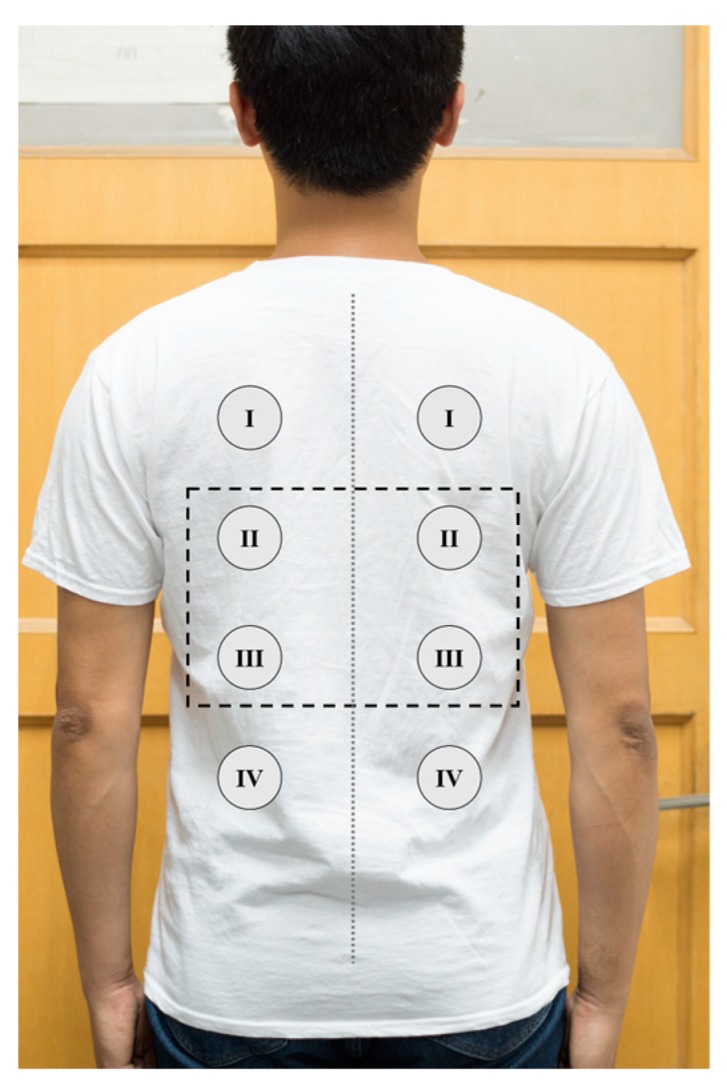
Different electrode positions.

There is no standard location for human back ECG signal measurement [30]. Various unipolar electrode positions are experimentally tested. With the spine as the center, the human back is divided into eight different positions, and the eight electrode positions are placed symmetrically on the left and right sides of the human back. These electrode positions are shown in Figure 10. One-channel unipolar ECG signals are obtained from five subjects (ages 22–32). One unipolar electrode is used to measure the ECG signal at these electrode positions. All the subjects wear uniform cotton T-shirt, and the thickness of the cotton T-shirt between the electrode and the skin is 0.3 mm. Using the amplitude of the QRS peak as the indicator for the sensitivity, the measurement results are listed in Table 1. It can be seen that the position I and II produce higher sensitivity than other positions. The position of the four unipolar electrodes (Figure 3a) is determined according to these experimental results.

### 3.2. The Influence of Clothing Thickness

**Figure 11:**
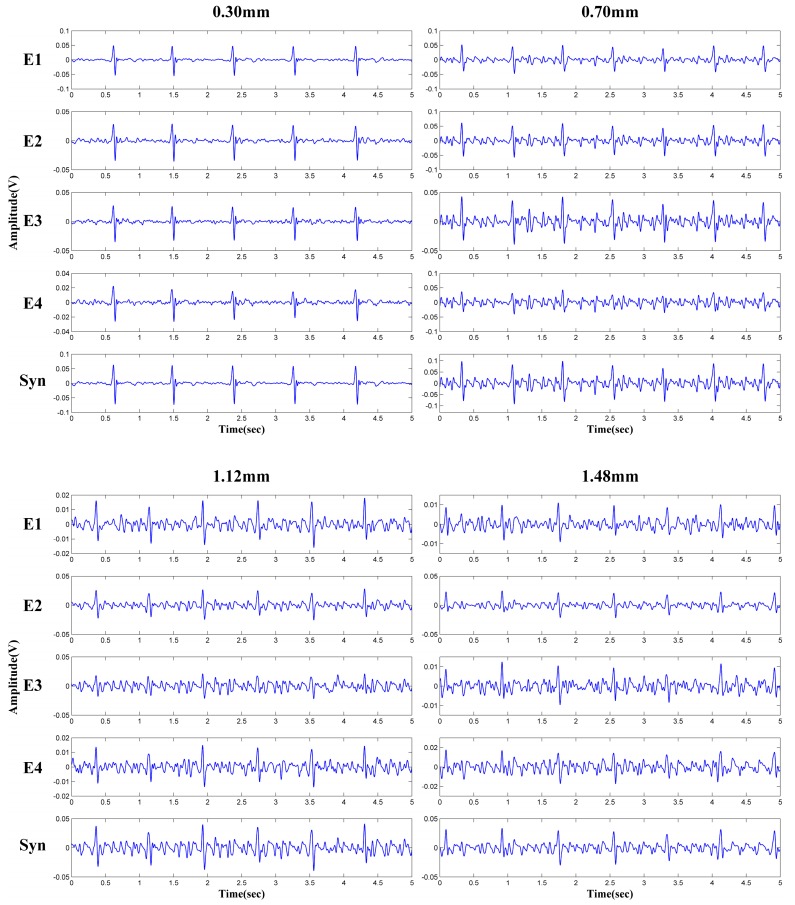
ECG signals under different clothing thickness.

The main advantage of the capacitively coupled electrode is the ability to work through insulation materials, such as fabric and clothing. In daily life, the subjects may wear clothes of different thickness. The influences of various clothing thickness on the system performance are evaluated. One subject (male, 1.75 m 76 Kg) is asked to wear different cotton T-shirt layers, and sit on the proposed ECG signal acquisition system. The thickness of the single-layer cotton T-shirt is about 0.3 mm. The multi-channel ECG signals are measured under different layers of cotton T-shirt. Figure 11 shows the measured multi-channel ECG signals and the synthetic single-channel results. The ECG signals of all channels are generally clear in case of the single-layer cotton T-shirt (0.3 mm). With the increasing of the clothing thickness, the ECG signal quality is declined. However, even in the case of 1.48 mm clothing thickness (four layers of cotton T-shirt), clear QRS complex features can still be observed.

### 3.3. Performance Tests

**Figure 12:**
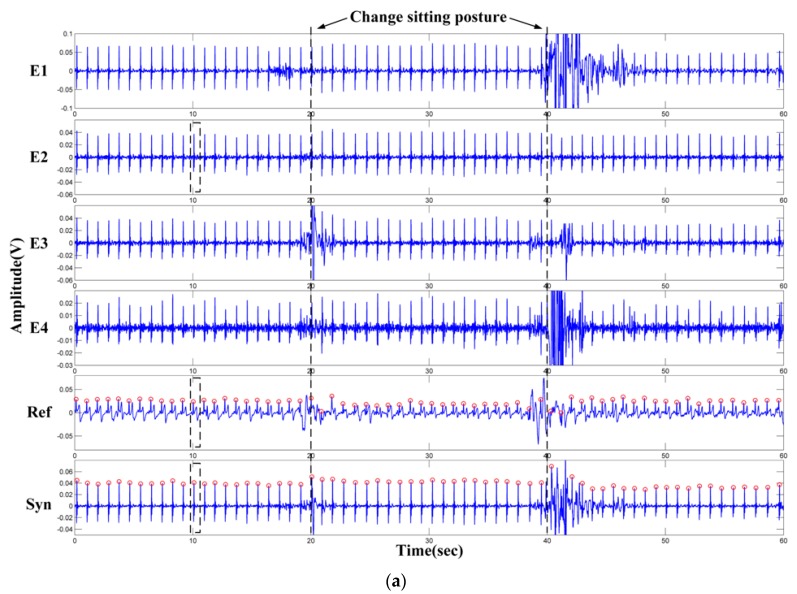

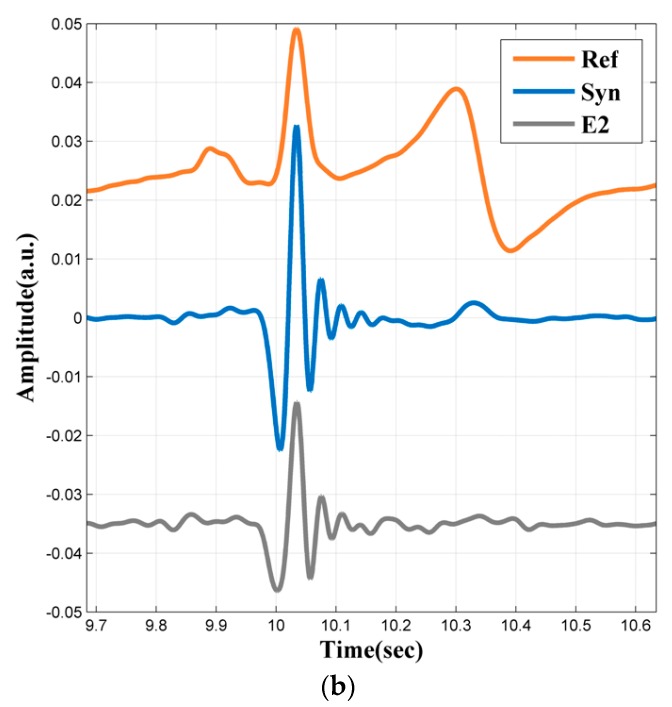
One-minute measurement results: (**a**) The measured ECG signals and the synthetic results; (**b**) Details of the ECG waveforms (near 10 s).

A healthy subject (male, 1.70 m, 68 kg, 0.3 mm thickness cotton T-shirt) was experimentally tested. The subject was asked to sit on the proposed ECG signal acquisition system. To measure the standard lead-I ECG signals, the LA, RA and LL extremity electrodes of the clinical ECG instrument (SE-601B, twelve-lead/channel EDAN Instruments, Shenzhen, China) are placed on the subject’s left arm, right arm and left leg respectively. One minute of ECG signals are simultaneously measured by our device and the clinical ECG instrument. The ECG signals obtained by the clinical ECG instrument are used as the reference ECG signals. During the experiment, the subject is asked to change the sitting posture at the 20 s and 40 s. The recorded ECG signals are shown in Figure 12a. The recorded signals are processed offline in Matlab to verify the validation of the above-mentioned signal processing method. The synthetic results are calculated every 10 s. As can be seen from Figure 12a, the changing in sitting posture has a great influence on the quality of the measured signal. Even for the standard lead-I ECG signals (Ref) measured by the clinical ECG instrument, motion artifacts still exist around the 20 s and 40 s. As for the synthetic results (Syn), when the motion artifacts are small (near the 20 s), relatively satisfied results can be obtained. However, the synthetic results are not stable when the motion artifacts are large and occur in most channels (near the 40 s). Adjusting the scaling factor σ in Equation (7) (e.g., increasing the weight value of E2) can improve the signal quality of the synthetic results. One heart beat cycle near 10 s (black rectangular frames in Figure 12a) is shown in Figure 12b. The QRS complex can be clearly observed by all the three ECG segments, and the T-wave can also be observed from the synthetic results. However, the P-wave is not obvious in the synthetic results and the measured ECG signals of E2. The waveforms of the standard lead-I ECG signals and the synthetic results are smoother than that of the E2.

Six subjects were asked to sit on the proposed ECG signal acquisition system and their ECG signals were measured within three minutes. During the experiment, the slight movements (typing, talking etc.) are allowed. All of the subjects are different in body shape and wear clothes of different thickness. The body mass index (BMI) is used to indicate their body shape. In the meantime, the standard lead-I ECG signals measured by the clinical ECG instrument are recorded as the reference ECG signals. One student with moderate training in ECG analysis annotated these measured ECG signals. The heart beat measurement results are listed in Table 2. Where TB is the total beats, TP is correctly detected beats (true positive), FN is undetected beats (false negative) and FP is falsely detected beats (false positive). The overall sensitivity (Se) and predictability (P) are 99.13% and 99.28% respectively.

## 4. Conclusions

A prototype of an ECG signal acquisition system with multiple capacitively coupled unipolar electrodes is described. Compared with those differential measurement approaches, in the proposed system, the ECG signals are directly obtained from the outputs of each unipolar electrodes. Because the measured signal from one sensing electrode is affected by the electrode-skin coupling impedance, the differential ECG signal obtained from outputs of one pair of electrodes may be ruined by the impedance imbalance. This is especially true when the ECG signal is measured in a capacitive-coupled way. On the other hand, since the signals are amplified and sampled separately in unipolar approach, problems caused by such imbalance can be suppressed. Experiments are carried out with four unipolar electrodes attached on the backrest of a chair. The reference electrode is mounted on the seat of the chair. The measurement ECG signals are likely to be less affected by its location. One important phenomenon is that the signal quality is affected by the softness of the backrest. An index for quality evaluation is proposed based on phase space reconstruction. Using the index as the weight value for weighted synthesis, a fast signal synthetic method is designed for obtaining a single-channel ECG signal from the multiple-channel unipolar signals. Although the obtained ECG signal quality is not higher than that by the differential measurement, the experimental results indicate that available ECG signals can be obtained in case of slightly human body motions. Future work will be aimed at the long-term evaluation under real-life conditions and new measurement configurations.

## Figures and Tables

**Table 1:** QRS peak amplitude results.

Left Electrode Position	QRS Peak Amplitude (Mean ± SD mV)	Right Electrode Position	QRS Peak Amplitude (Mean ± SD mV)
I	71.58 ± 5.38	I	80.63 ± 4.20
II	68.93 ± 4.51	II	74.97 ± 5.22
III	34.04 ± 3.93	III	37.55 ± 4.71
IV	24.35 ± 3.23	IV	21.54 ± 3.97

**Table 2:** Heart beat measurement results.

Sub. (BMI [kg/m^2^], Cloth Thickness [mm])	TB	TP	FN	FP	Mean HR ± SD (bpm)	Se (%)	P (%)
No. 1 (23.60, 0.30)	198	184	4	3	66.0 ± 1.0	97.87	98.40
No. 2 (25.34, 0.35)	222	219	3	0	74.0 ± 3.0	98.65	100.00
No. 3 (24.82, 0.30)	196	194	2	3	65.3 ± 2.3	98.98	98.48
No. 4 (24.80, 0.26)	214	213	1	0	71.33 ± 1.2	99.53	100.00
No. 5 (25.63, 0.55)	217	215	1	2	72.3 ± 0.6	99.54	99.08
No. 6 (19.38, 0.40)	224	224	0	1	74.7 ± 2.5	100.00	99.56
Total	1271	1249	11	9	-	99.13	99.28
